# Supplementary material for: Evidence supported by Mendelian randomization: impact on inflammatory factors in knee osteoarthritis
Source: Front Med (Lausanne). 2024 May 28;11:1382836. doi: 10.3389/fmed.2024.1382836 (PMC11165061; doi:10.3389/fmed.2024.1382836)
Supplement: Supplementary file 1 [file Data_Sheet_1.ZIP › data/GCST90274794_5e6/794.leaveoneout.pdf]

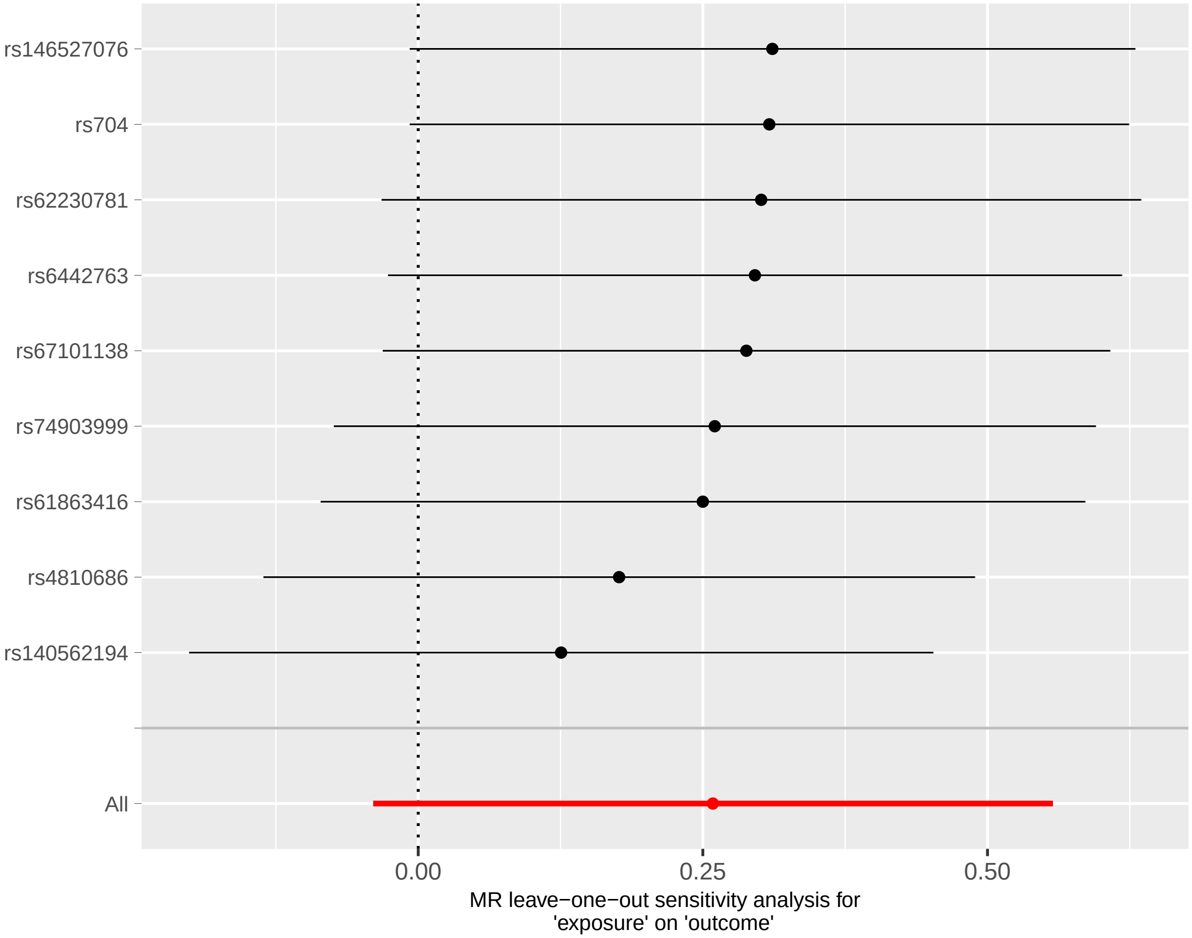

rs146527076
rs704
rs62230781
rs6442763
rs67101138
rs74903999
rs61863416
rs4810686
rs140562194
All
0.00
0.25
0.50
MR leave−one−out sensitivity analysis for
'exposure' on 'outcome'
